# Identification of a Novel Regulator for the *Escherichia coli fit* Iron Transport System

**DOI:** 10.2174/1874285800802010094

**Published:** 2008-07-08

**Authors:** Zhiming Ouyang, Richard Isaacson

**Affiliations:** 1 Department of Microbiology, University of Texas Southwestern Medical Center, Dallas, Texas USA; * Department of Veterinary and Biomedical Science, University of Minnesota, ST PAUL, MN, USA

## Abstract

The *Escherichia coli fit* iron transport system consists of 6 genes, *fitA, B, C, D, E* and *fitR*. Based on* in silico* analysis, FitA-E composes a typical bacterial iron transporter, while FitR was deduced to be a regulator. In this paper the regulation of *fit* expression by FitR was studied using a quantitative RT-PCR technique and a *lacZ* reporter assay. It was found that *fit* expression was repressed when FitR was over-expressed and de-repressed when *fitR *was knocked out by mutation. When the mutation in* fitR* was complemented in *trans-* with the wild type *fitR* gene, repression of *fit* expression by FitR was restored. Finally, recombinant FitR was found to bind to the* fit* promoter DNA when employed in an electrophoretic mobility-shift assay. These results demonstrated that *fitR* encodes an auto-repressor for the *E. coli* *fit *system.

## INTRODUCTION

Iron is an essential element for bacterial growth and pathogenesis. Although there is sufficient iron in the environment, iron is not readily available to bacteria in animal or human hosts [1]. To support growth, bacteria have evolved to possess multiple iron transport systems to acquire iron from the environment or hosts. In a previous study, a novel putative ABC iron transport system, *fit*, was identified in an *E. coli* strain causing human septicemia [2]. The* E. coli* *fit* system consists of 6 genes designated *fitA, -B, -C, -D, -E* and *fitR*, encoding an outer-membrane receptor protein (FitA), a periplasmic binding protein (FitE), two permease proteins (FitC and -D), an ATPase (FitB), and a hypothetical protein (FitR) [2]. Gene *fitA,-B, -C, -D, -E* encode a typical bacterial iron transporter. Although there is no match for the predicted FitR protein through BLAST analysis, sequence analysis found that an N-terminal helix-turn-helix DNA binding motif and a C-terminal sugar isomerase domain were present in the predicted FitR. This structure is conserved in many bacterial transcriptional regulators, such as *E. coli* RpiR, LpcA, GutQ, and Glms [3, 4]. In this paper, quantitative RT-PCR and a *fit-lacZ* reporter analysis were employed to study the expression of *fit* system under conditions of *fitR* over-expression, and mutation and complementation of *fitR*. It was found that *fitR* repressed *fit* expression.

## METHODS

### Bacterial Strains and Media

The bacterial strains and plasmids used in this study are listed in Table **1**. All strains were grown in Luria-Bertani (LB) media. All reagents and media were made with deionized water after passage through a Millipore Catridge system (Millipore, USA). All glassware was treated with 8M HCl and then rinsed 3 times with double distilled H_2_O. When appropriate, supplements were added to media at following concentrations: ampicillin, 100 μg/ml; tetracycline, 15 μg/ml; kanamycin, 50 μg/ml; trimethoprim, 100 μg/ml.

### Recombinant DNA Techniques

General genetic techniques including PCR, genomic or plasmid DNA purification, ligation and transformation were performed as described previously [2]. Restriction endonuclease and DNA-modifying enzymes were used according to the manufacturer’s recommendations. DNA fragments were purified from agarose gels using Qiaquick gel extraction kit (Qiagen, USA). DNA cloning and manipulation were conducted in *E.coli* DH5*α* cells. All oligonucleotide primers were commercially synthesized by IDT (Integrated DNA Technologies, USA) and listed in Table **2**. DNA sequencing work was done at the Advanced Genetic Analysis Center (AGAC) at the University of Minnesota.

### Construction of Transposon-Insertional Mutants

The EZ::TN transposons (Epicentre, Madison, Wisconsin, USA) were used to create gene disruption mutants in *E. coli* i484 through i*n vitro* transposon insertional mutagenesis according to the protocols provided by the manufacturer [5, 6]. Briefly, the particular gene to be mutated was amplified by PCR. After purification, an EZ::TN transposon was inserted into the DNA fragment through *in vitro* transposition using the EZ::TN transposase. The disrupted DNA was then electroporated as linear DNA into a recipient strain containing the plasmid pKD46 [5]. Mutants were screened for antibiotic resistance encoded on the inserted EZ::TN transposon and the presence of the transposon in the mutated gene was confirmed by PCR amplification and DNA sequence analysis. Plasmid pKD46 was then eliminated from these cells by growth at 42^o^C.

### RNA Extraction and Real-Time RT-PCR Analysis

Bacteria were grown in LB with appropriate supplements at 37°C with shaking. After 3 hours, cells were harvested, lysed and RNA was isolated using the Qiagen RNeasy Mini Kit (Qiagen, USA). Reverse transcription was performed using the SuperScript™ III Reverse Transcriptase (Invitrogen, USA). Real-time PCR (qPCR) using Platinum® SYBR® Green qPCR SuperMix-UDG (Invitrogen, USA) was then performed to measure the level of gene expression. The genes *gst* and *polA* were used as endogenous controls.

### β-Galactosidase Assays

Bacteria carrying *lacZ* reporter vectors grown in appropriate media were harvested when bacterial growth reached exponential growth phase. Ortho-nitrophenyl-*β*-D-galacto-pyranoside (ONPG, Sigma, USA) was used as the substrate to measure *β*-Galactosidase activities and the Miller Units was calculated as described [7]. Each experiment was repeated 3 times, and the results were statistically analyzed using the Student’s *t* test.

### Construction of Tightly-Controlled FitR Expression Plasmid

To control expression of *fitR*, a *fitR* expression plasmid, pBAD-fitR1, was constructed by cloning the *fitR* gene into the expression vector pBAD22 [8]. Primers (listed in Table **2**) were used to PCR amplify* fitR* using high-fidelity DNA *pfu* polymerase (Stratagene, USA). The forward primer contained an *EcoR* I site, and the reverse primer contained a *Hind *III site. The fragment was cloned into pBAD22 cleaved with *EcoR *I and *Hind *III. Protein expression from pBAD-fitR1 was tightly controlled by the arabinose pBAD promoter and was induced by addition of arabinose to the growth medium.

### Cloning and Purification of FitR

A his-tagged *fitR* expression plasmid was constructed by cloning *fitR* into the expression vector pBAD22. A 6 × his tag sequence was included in the reverse primer, which produced a C-terminal 6 X his tag for protein purification (listed in supplemental materials Table **2**). Then the amplified fragment was digested with *EcoR* I and *Hind *III, and ligated into plasmid pBAD22. Positive clones were identified through PCR and sequence analysis. In order to express the recombinant Fur protein, DH5α cells carrying the positive *fitR* expression plasmid pBAD-fitR2 were grown in LB media. When bacterial growth reached an A_600_ of 0.6, arabinose was added to the culture at a final concentration of 10mM to induce protein expression. After 5 hours of induction, cells were harvested by centrifugation (4000g for 20min at 4°C). Protein was purified by Ni-nitrilotriacetic acid (NTA) affinity chromatography, using Ni-NTA spin columns (Qiagen, USA), under denaturing conditions according to the manufacturer's instructions. Then the purified FitR was refolded by dialysis against 10mM Tris (pH 7.0). After the pellet was removed by centrifugation at 10,000 rpm for 10min, supernatant was collected, analyzed by SDS-polyacrylamide gel electrophoresis, quantified using the Bradford assay kit (Bio-Rad, USA) and used as active FitR in the following assays.

### Electrophoretic Mobility-Shift Assay

The electrophoretic mobility-shift assay was performed as follows. The 347bp intergenic region between *fitA* and *fitB*, including the *fit* promoter, was PCR amplified using *pfu* polymerase (Stratagene, USA) with synthesized primers (listed in Table **2**). The purified DNA was end-labeled with digoxigenin using recombinant terminal transferase (Roche Applied Science, USA). Three nanograms of labeled DNA and various amounts of purified FitR protein were mixed and incubated at 37°C for 20min. The standard binding buffer was: 20mM Bis-Tris (pH 7.0), 2.5µg/ml sonicated salmon sperm DNA, 50μg/ml poly[d(I-C)], 5%(w/v) glycerol, 100µg/ml BSA, 1mM MgCl_2_, 40mM KCl and 100µM Mn^2+^. Protein-DNA complexes were separated from unbound DNA on 5% nondenaturing polyacrylamide gels run at 50V for 2 to 3h. The DNA was transferred onto a positively charged Nylon membrane (Roche Applied Science, USA) by electroblotting. The digoxigenin-labeled probes were subsequently detected by an enzyme immunoassay using an antibody (anti-digoxigenin-AP, Fab fragments) and the chemiluminescent substrate disodium 3-(4-methoxyspiro {l,2-dioxetane-3,2'-(5'-chloro)tricyclo[3.3.1.1^3,7^]decan}-4-yl) phenyl phosphate (CSPD) (Roche Applied Science, USA).

## RESULTS AND DISCUSSION

### FitR Represses *fit *Expression

**Fig. (1):**
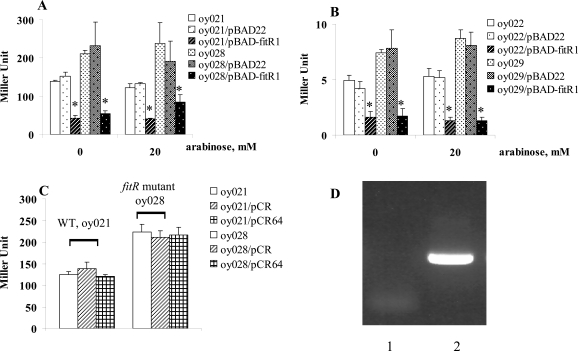
FitR repressed *fit* expression, measured by *lacZ* reporter assays. Strains carrying *pMP-fitA* (A) or* pMP-fitB* (B) were grown in LB. When bacterial growth reached an A600 of 0.5-1.0, cells were harvested and *β*-galactosidase activities were measured as described (13). Y axis represents LacZ level, indicated in Miller Units. Star (* ) represents statistically significant (p < 0.05).

A *lacZ* reporter assay was employed to determine if FitR regulated *fit* expression. The intergenic DNA containing the bidirectional *fit* promoter, located between *fitA* and *fitB*, was cloned in two directions into the *lacZ* reporter vector, pMP220. The two *fit-lacZ* reporter plasmids were named pMP-fitA and pMP-fitB, and were used to measure the expression of *fitA* and *fitB*, respectively [2]. *β* -galactosidase activities were measured from exponential growth phase cells as described [7]. As shown in Fig. (**1**), the expression of *fitB* (Fig. **1A**) in strains containing pMP-fitB was very similar to the strain carrying pMP-fitB and the cloning vector pBAD22. The wild type strain (oy021) containing the plasmid pMP-fitB expressed 139 units of *β*-galactosidase (Fig. 1A). When FitR was expressed from pBAD-fitR1 in wild type strains, this strain expressed 43 units of *β*-galactosidase, which showed that expression of *fitB* was repressed 3.2 fold. Further, cells expressed 211 units of *β*-galactosidase in the *fitR* knockout mutant, indicating that *fitB *was de-repressed 4.9 fold in the* fitR *mutant (oy028).**Finally,**when the *fitR* mutation was complemented in *trans-* by pBAD-fitR1, cells expressed 55 units of *β*-galactosidase, which confirmed the observation that *fit *was repressed by FitR. A similar regulation pattern was obtained for strains containing pMP-fitA (Fig. **1B**). These data demonstrated that FitR does repress *fit* expression.

The repression of *fit* by FitR was also found even when FitR expression was not induced (0mM arabinose, Fig. **1**), which might be the result of leaky expression of *fitR* from pBAD-fitR1. To test this, we cloned *fitR* into the plasmid pCR-XL-TOPO, a cloning vector without a ribosome binding site (RBS). The new plasmid was designated pCR64. When this plasmid was introduced into wild type strains oy021 or *fitR* mutant oy028, strains oy021/pCR-XL-TOPO, oy021/pCR64, oy028/pCR-XL-TOPO and oy028/pCR64 expressed 129, 120, 228 and 217 units of *β*-galactosidase, respectively, which demonstrated that *fit* expression was not repressed without *fitR* expression (Fig. **1C**). Further, we examined *fitR* transcription using RT-PCR. As shown in Fig. (**1D**), the *fitR* transcript was detected in cells carrying pBAD-fitR1 grown under non-induced condition (lane 2). Thus we concluded that the repression of *fit* by pBAD-fitR1 when cells were grown in LB containing 0mM arabinose resulted from the leaky expression of FitR from pBAD promoter. The fact that pBAD22 is quite leaky has been found in other studies [8, 9]. The regulation of the *fit *system by FitR was also examined using a quantitative RT-PCR analysis. Compared to gene expression in wild type strain *E. coli* i484, *fitB* was repressed 1.8 fold when FitR was expressed from pBAD-fitR1 and de-repressed 3.2 fold when *fitR* was mutated. In addition, when the *fitR* mutation was complemented by pBAD-fitR1, *fitB* was repressed 6.8 fold compared with gene expression levels in the* fitR* mutant. Similar results were obtained for *fitA *expression. These results demonstrated that FitR represses the expression of the *fit* system.

### Binding of Recombinant FitR to the *fit *Promoter

If FitR directly regulates the *fit* system, it would be expected to bind to the *fit* promotor. To obtain protein FitR, a *fitR* over-expression plasmid, pBAD-fitR2, was constructed by cloning *fitR* into plasmid pBAD22. *E. coli* DH5α cells carrying pBAD-fitR2 were grown in LB and protein expression was induced by 10mM arabinose. As shown in Fig. (**2A**), the purified recombinant FitR was found to have a molecular weight of 28kDa (lane 1), which is consistent with the predicted molecular weight based on sequence analysis. To determine if FitR bound to the *fit* promoter, an electrophoretic mobility-shift assay (EMSA) was performed. As shown in Fig. (**2B**), the purified FitR was found to bind to the *fit* promoter DNA when used at 20, 50, or 100ng (lane 2, 3, 4, 5). When a 50 fold excess of non-labeled *fit* promoter DNA was used as a specific competitor in the reaction, the binding of FitR was found to be inhibited (lane 6). These results suggested that FitR binds specifically to the *fit *promoter.

### *Fit*R Repression of the *fit* System was Independent of *Fur’*s Regulation

**Fig. (3):**
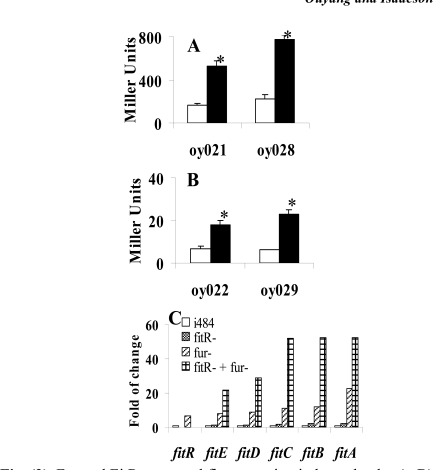
Fur and FitR repressed fit expression independently. A, B), Iron is not required for repression of fit expression by FitR.  *E. coli*strains carrying pMP-fitB (A) or pMP-fitA (B) were grown in LB (open bar) or LB with 100µM 2, 2’-dipyridyl (filled bar). Star (* ) represents statistical significant (p<0.05). C), Gene expression levels measured using quantitative RT-PCR analysis. RNA was isolated from bacteria grown in LB for 3 hours. Strains used were:  *E. coli * i484; oy071 (i484, *fur::*DHFR-1), oy077 (*fitR::*kan-2), and oy078 (*fur* and *fitR* double mutant). Results were shown as fold of change: ratio of gene expression levels under other conditions to the expression level in * E. coli * i484 (open bar). Values are the means from three independent experiments.

The *E. coli* *fit* system has been found to be repressed by Fur-Fe^2+^ complex (Ouyang and Isaacson unpublished). Therefore, *fit* is regulated by two repressors: Fur and FitR. Here we investigated the possibility that these two repressors regulated *fit* expression independently. Initially, a *lacZ* reporter assay was performed to determine if FitR needed iron as a cofactor to repress *fit* expression. As shown in Fig. (**3A**), the wild type strain containing pMP-fitB (oy021) expressed 168 and 525 units of *β*-galactosidase under iron-rich and iron-deficient conditions, respectively. In the *fitR* mutant (oy028), cells expressed 227 and 777 units of *β*-galacto-sidase under iron-rich and iron-deficient conditions, respectively. Similar results were obtained for pMP-fitA (Fig. **3B**). These results indicated that *fit* expression was still induced by iron deprivation when *fitR* was mutated, which in turn suggested that iron and FitR separately repressed *fit* expression. In addition, a quantitative RT-PCR analysis was employed to study if the repression of FitR is independent of expression of Fur. As shown in Fig. (**3C**),* fitA* expression was induced 23 fold in the *fur* mutant, 2.2 fold in the *fitR* mutant, and 52 fold in the *fitR *and *fur* double mutant. These data suggested that FitR**and**Fur repressed *fitA* independently. Similar results were observed for other* fit* genes.

In *E. coli*, for those well-studied iron transporters, such as *fep*, *fhu*, *fec*, *feo*, they are all regulated by the global regulator, Fur (ferric uptake regulator), through binding of Fur-Fe^2+^ complex to the Fur box in the gene promoters [10, 11, 12]. And except for the *fec* system, most *E. coli* iron transporters do not have their own regulators [13, 14, 15, 16]. Interestingly, the *E. coli fit* system is regulated by two independent repressors, an auto-regulator FitR and a global regulator Fur. This elegant regulation may indicate the important role of *fit* in *E. coli* metabolism. So far we don’t know if FitR is a global regulator or if any other factor is involved in the regulation of the *fit* system. Further studies, for example, identification of the exact binding site of FitR or Fur on *fit* promoter, whether there is a synergy in binding by addition of Fur and FitR, and whether other proteins might be involved in the expression of *fit*, etc, may elucidate the detailed regulation mechanism of this novel iron transporter.

## CONCLUSION

In conclusion, an auto-repressor, FitR, was identified for the *E. coli fit* iron transport system in this report.

## Figures and Tables

**Fig. (2):**
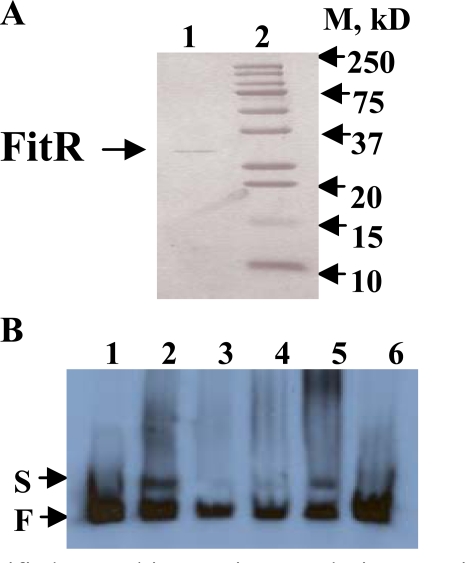
A), Purified recombinant His-tagged FitR protein analyzed by a 15% SDS-PAGE gel. Lane1, His-tagged FitR; lane 2, standard protein Marker. B), FitR binds to the *fit* promoter. All the lanes contained 3ng end-labeled *fit* promoter DNA. Lane 1 contained no protein; lanes 2-5 contained 100, 10, 20, and 50ng purified FitR, respectively. Lane 6 contained 50ng FitR and 150ng unlabeled *fit* promoter DNA. S and F indicate FitR bound and free DNA bands, respectively.

**Table 1.** Bacterial Strains and Plasmids Used in this Study

Strain and Plasmid	Deskription	Reference
***E. coli* strains**		
i484	O25:H autoagglutinating, human isolate	17
oy077	i484, *fitR* :: EZ-TN5< Kan-2 >	This study
oy016	i484, *lacZ* :: EZ-TN5< DHFR-1 >	This study
oy021	oy016 carrying pMP-fitB	This study
oy022	oy016 carrying pMP-fitA	This study
oy026	oy016, *fitR* :: EZ-TN5< Kan-2>	This study
oy028	oy026 carrying pMP-fitB	This study
oy029	oy026 carrying pMP-fitA	This study
oy071	i484, *fur* :: EZ-TN5 <DHFR>	This study
oy078	oy077, *fur* :: EZ-TN5<DHFR>	This study
DH5α	*F–, ф80d lacZ∆M15 ∆ (lacZYA–argF) U169, endA1, recA1, hsdS17 (rK–mK+), deoR, thi–1, supE44, λ–, gyrA96, relA1*	Invitrogen
		
**Plasmids**		
pMP220	Contains a promoterless *lacZ* gene, Tet^r^	19
pMP–*fitA*	*fit* promoter was cloned into pMP220 with a direction of *fitA* transcription	2
pMP–*fitB*	*fit* promoter was cloned into pMP220 with a direction of *fitB* transcription	2
pBAD22	overexpression vector, Amp^r^	8
pBAD–fitR1	DNA fitR1 cloned into pBAD22 at * EcoR *I and *Hind *III sites	This study
pBAD–fitR2	DNA**fitR2 cloned into pBAD22 at *EcoR *I and *Hind *III sites	This study
pCR	Cloning vector pCR-XL-TOPO	invitrogen
pCR64	DNA fitR1 cloned into pCR-XL-TOPO	This study

**Table 2.** Oligonucleotide Primers Used in this Study

	Gene	Primer, 5’-3’	
PCR and cloning			
	*fitR1*	forward	CGGAATTCGCGAGTCAGTTAACGCCT
	* *	reverse	TATAAGCTTGCCATTCTTTCTTCCCGT
	*fitR2*	forward	CGGAATTCATGATCCAGAAAGATAAAGTGCG
	* *	Reverse	TATAAGCTTTTA**GTGATGGTGATGGTGATG**GCCATTCTTTCTTCC
	*fit promoter*	forward	GTGCCACGTGTACGCCAACTTAAT
		reverse	TCGCGGATTGTCGCGAGTGAAA
			
qPCR			
	*fitA*	forward	ACCATGCTCTATTCCCATCG
		reverse	GGCTGTTTCGTCGTAAGGTC
	*fitB*	forward	GCACCATTGCACGTATCTTG
	* *	reverse	ATAACGCTTTCGTTGGTTGC
	*fitC*	forward	ACCTTGCACCCTCCCTTAAC
	* *	reverse	CAAGCAGGGTAAATGTTCGC
	*fitD*	forward	TCTGCTTGGTCATCAGGTTG
	* *	reverse	AACAACGCCAGACGAGAACT
	*fitE*	forward	TATCGGTGATTCAGGCAAACCAGG
	* *	reverse	GTAATACCCGTCCCAGCGAATGAT
	*fitR*	forward	ATGAGCATCGTGAAGTGGTACTCG
	* *	reverse	ATAGCCCTGATAACTGTGGCATCG
	*fepA*	forward	GCCGATTGATTTCCTCGTAA
	* *	reverse	ATCCGTTGCTGATTAAACTA
	*gst*	forward	GAAGCTGCAATATGTGAACGAGGC
	* *	reverse	AGCGCAGAACCGTAAACAGATAGG
	*polA*	forward	TGAGTTCAAACGCTGGACTG
		reverse	TCTGCAACACTGGTTTCCTG

Restriction enzyme sites were depicted as underlined. In the reverse primer for *fitR2*, the 6 x his tag sequence was indicated in bold.
